# Construction and simulation-experimental characterization of a fluorescence image-based finite element model for cell mechanics

**DOI:** 10.3389/fbioe.2026.1894529

**Published:** 2026-07-22

**Authors:** Danyang Zhang, Shaodong Wu, Simian Zhu, Zhu Zeng

**Affiliations:** 1 School of Biology and Engineering (School of Modern Industry for Health and Medicine), Guizhou Medical University, Guiyang, China; 2 Logistics Support and Equipment Department, Shanghai Children’s Medical Center GuiZhou Hospital, Shanghai Jiao Tong University School of Medicine, Guiyang, China; 3 Centre of Cellular Immunotherapy Engineering Technology of Guizhou, Guiyang, China

**Keywords:** cell mechanics modeling, cytoskeleton, Young’s modulus, image processing, finite element analysis

## Abstract

**Objective:**

A finite element (FE) model was developed to characterize the three-dimensional (3D) heterogeneous structure features of cells, and the influence mechanism of cytoskeleton remodeling on the mechanical properties of cells and the intracellular stress transmission behavior was analyzed.

**Methods:**

Human lung adenocarcinoma A549 cells were used in this study, and the cytoskeleton model was constructed by using cytochalasin D (Cyto-D) at concentrations of 0, 0.1, and 1.0 μmol/L. Based on the 3D fluorescence image stacks obtained by confocal laser scanning microscopy (CLSM), combined with the image processing algorithm written in MATLAB, the 3D surface structure of the cells was reconstructed. Atomic force microscopy (AFM) was used to perform nanoindentation experiments to characterize the mechanical parameters of the cells. A multi-body contact FE model of “probe-cytoskeleton-nucleus-culture dish” was constructed based on the ANSYS platform, and the stress distribution and mechanical response of cells under indentation loading were simulated on this basis.

**Results:**

With the increase of Cyto-D concentration, the AFM nanoindentation showed that the cell height significantly decreased, while the Young’s modulus and adhesion force increased (*p* < 0.05). The FE simulation showed an approximately linear stress-strain relationship during the probe displacement loading. The image-based cell models exhibited obvious structural dependence, demonstrating non-uniform and asymmetric mechanical transmission. The deformation, strain, and stress levels of the nucleus were lower than those of the cytoskeleton region, but it still participated in progressive force transmission, forming a continuous gradient. There was no abrupt mechanical response at the nucleus-cytoskeleton interface, but a smooth transition, indicating that this interface had a certain buffering effect on the load and helped maintain the stability of the internal structure of the cells. With the increase of Cyto-D concentration, the simulation results of the model changed from local concentration to directional expansion and finally developed into a diffuse distribution. These findings suggest that the cytoskeleton disassembly process dominated the changes in the mechanical behavior of the cells.

**Conclusion:**

The proposed framework provides a feasible approach for integrating fluorescence imaging with FE analysis in cell mechanics research and may serve as a methodological reference for studying the relationship between cytoskeletal organization and cellular mechanical behavior.

## 1 Introduction

Cells are the fundamental structural and functional units of life, and their mechanical properties, including rigidity, adhesion, and viscoelasticity, not only maintain cellular morphological stability but also play important regulatory roles in migration, differentiation, and signal transduction (Xi et al., 2018). During physiological processes, cells generate endogenous active stress while being subjected to passive mechanical stimulation from the extracellular microenvironment (Efremov et al., 2021; Zhu et al., 2000). Quantitative studies on the mechanical properties of cells, such as deformation, rigidity, and viscoelasticity, as well as the mechanisms by which mechanical factors influence activities such as proliferation, migration, and differentiation, have always been core topics in the field of cell mechanics (Gómez-González et al., 2020; Ingber, 2023; Bashirzadeh and Liu, 2019; Chowdhury et al., 2021).

The mechanical behavior of living cells is mainly controlled by the cytoskeletal network, which is composed of microfilaments, microtubules, and intermediate filaments (Sun et al., 2023). This dynamic network plays a vital role in maintaining cell integrity, mediating intracellular transport, driving cell movement, resisting deformation, and adapting to morphological changes (Ingber et al., 2014; Wendling et al., 2003; Fletcher and Mullins, 2010). For instance, fluid shear stress on endothelial cells can enhance cellular rigidity by inducing cytoskeletal rearrangement through the activation of hormone release and intracellular calcium signaling (Sato et al., 1987; Wakatsuki et al., 2001).

Confocal laser scanning microscopy (CLSM) enables 3D high-resolution and multi-channel imaging of fluorescently labeled living cells or specific proteins (Bourn et al., 2025; Markov et al., 2021). Atomic force microscopy (AFM) can simultaneously obtain cell morphology images and measure their mechanical properties under physiological conditions (Viji Babu and Radmacher, 2019; Zhou et al., 2021; Dufrêne et al., 2017). Traditional analytical models (such as the Hertz contact model) can correlate the applied force and indentation depth with Young’s modulus, but they require the assumption that the contacting bodies are semi-infinite, homogeneous, and continuous with smooth surfaces (Lim et al., 2006; Hertz, 1882). As a complementary tool to experimental research, finite element (FE) analysis has played a significant role in the development of cell mechanics (Kasas et al., 2004).

However, current cell-level FE analysis still faces two major bottlenecks. The first challenge is insufficient geometric fidelity. Although CLSM can provide high-resolution 3D fluorescence data, converting these data into meshes suitable for FE analysis is time-consuming, labor-intensive, and has limited automation due to issues such as imaging noise, uneven labeling, and blurred boundaries (Dufaux, 2021; Zhang, 2012; Liu et al., 2019). Therefore, most models rely on highly idealized geometric shapes (such as uniform half-spaces, spheres, or ellipsoids) and isotropic linear elastic or viscoelastic constitutive models, ignoring prestress, anisotropy, and subcellular heterogeneity (Zhou et al., 2005; Milner et al., 2012; Barreto et al., 2014). This limitation reduces the prediction accuracy of intracellular stress transmission (Wang et al., 2009; Murphy et al., 2014). Second, the computational efficiency is relatively low. The initial STL surface models reconstructed from CLSM images often contain topological defects such as non-manifold edges, holes, self-intersections, and fragmentation, which can easily lead to mesh generation failure or solver convergence failure when directly used in FE analysis (Chen and Giannone, 2024; Vasan et al., 2020).

**FIGURE 1:**
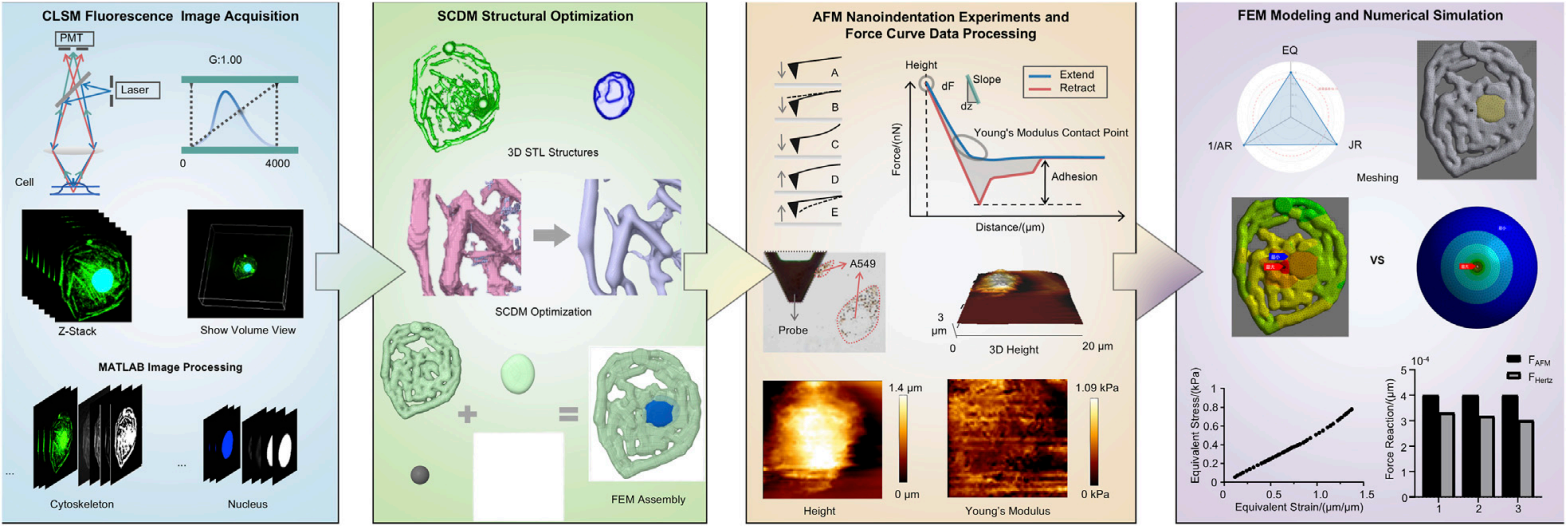
Overall technical workflow.

In this study, 3D fluorescence image stacks of A549 cells were acquired using CLSM, and high-fidelity STL geometries of the cell and nucleus were reconstructed using an in-house MATLAB-based image-processing algorithm. Topological repair and geometric optimization were subsequently performed in the SpaceClaim Direct Modeler (SCDM) environment to improve FE convergence for complex biological structures. The quantitative imaging (QI) mode of AFM was then employed to obtain mechanical parameters for A549 cells. Based on these data, a multibody contact FE model consisting of the probe, cytoskeleton, nucleus, and culture dish was established. The numerical framework was parameterized using experimentally measured mechanical properties, and intracellular stress distributions under indentation loading were further analyzed. The overall technical route is shown in Figure 1. Compared with conventional cell FE models based on idealized geometries or manually reconstructed structures, the present framework combines fluorescence image-derived 3D reconstruction, automated image processing, topology optimization, and finite element simulation within a unified workflow. This approach preserves cell-specific structural heterogeneity while improving model robustness and computational convergence.

## 2 Materials and methods

### 2.1 Reagents and equipment

RPMI-1640 medium (Thermo Fisher Scientific, United States); fetal bovine serum (FBS) (Tianhang Biotechnology, Zhejiang, China); phosphate-buffered saline (PBS) (Macklin Biochemical Technology, Shanghai, China); bovine serum albumin (BSA) (Solarbio, Beijing, China); 4% paraformaldehyde (PFA) (Solarbio, Beijing, China); DAPI solution (Solarbio, Beijing, China); Alexa Fluor 488-conjugated phalloidin (UElandy, China); anti-fade mounting medium (Solarbio, Beijing, China); cytochalasin D (Cyto-D) (Macklin, Shanghai, China). All the reagents were used after purchase without any purifications.

CO_2_ cell culture incubator (Thermo Fisher Scientific, United States); electronic balance (Puchun Instruments, Shanghai, China); pH meter (Yueping Scientific Instruments, Shanghai, China); vertical autoclave (Shenan Medical Equipment, Shanghai, China); 4 °C/−20 °C refrigerator (Haier Special Electric Appliances, Qingdao, China); −80 °C vertical ultra-low temperature freezer (Haier Special Electric Appliances, Qingdao, China); benchtop high-speed centrifuge (Xiangyi Laboratory Instrument, Hunan, China); clean bench (AIRTECH, Suzhou, China); AFM (NanoWizard 4 XP, Bruker, Germany); workstation (ASUS, China; Intel Core i9-12900 KF CPU, 128 GB DDR4 RAM, NVIDIA Quadro RTX A4000 16 G GPU); CLSM (AX-SHR, Nikon, Japan) equipped with a LUA-S6 multi-line laser unit and mounted on an Eclipse Ti2-E inverted microscope (Nikon, Japan).

### 2.2 Cell culture and pharmacological treatment

The human lung adenocarcinoma cell line A549 was used in this study. Cryopreserved A549 cells were rapidly thawed in a 37 °C water bath, followed by centrifugation at 2,000 × g for 5 min. After removal of the supernatant, the cells were resuspended in RPMI-1640 complete medium, seeded into T25 culture flasks, and maintained in a humidified incubator at 37 °C with 5% CO_2_. Cell adhesion and growth were monitored daily, and the culture medium was replaced every 48 h. Cells were subcultured according to their growth conditions. Cells in the logarithmic growth phase were seeded into six-well plates or confocal culture dishes at a density of 3 × 10^5^ cells/mL. After overnight attachment and spreading, the cells were treated with Cyto-D for 1 h. Cells were divided into three groups: control (0 µM), low concentration (0.1 µM), and high concentration (1.0 µM).

### 2.3 Fluorescence staining of the cytoskeleton and nucleus

Following Cyto-D treatment, cells were fixed with 4% PFA at room temperature for 20 min, permeabilized with 0.1% Triton X-100 for 10 min, and blocked with 0.5% BSA for 30 min. The cytoskeleton was stained with Alexa Fluor 488-conjugated phalloidin (1:200 dilution) for 30 min in the dark, followed by nuclear counterstaining with DAPI for 10 min. Samples were washed with PBS between each step. Finally, antifade mounting medium was added, and the stained samples were stored at 4 °C in the dark before imaging.

### 2.4 CLSM fluorescence image acquisition

Stained samples were mounted on the stage, and ambient light was minimized to reduce stray light interference. Initial observation and positioning were performed using a low-magnification objective lens (×10 or 20×) at low resolution (512 × 512 pixels, zoom = 1.0) under transmitted bright-field or fluorescence imaging conditions to identify the imaging region and focal plane. Excitation wavelengths of 488 nm and 405 nm were selected. During multichannel acquisition, only the corresponding laser channel was activated to minimize spectral crosstalk. The pinhole diameter was set to 1 Airy unit to balance optical section thickness and signal-to-noise ratio. The photomultiplier tube (PMT) gain was adjusted according to the green fluorescence intensity to avoid signal saturation while maintaining sufficient contrast. High-resolution imaging was subsequently performed using a 60× or ×100 oil-immersion objective at a resolution of 1,024 × 1,024 pixels. The scanning speed was set to a moderate level, line averaging was set to 2, and the PMT gain was maintained below 800. Three randomly selected fields of view were acquired for each group. Under oil immersion, the zoom factor was fixed at 5. XYZ acquisition mode was used to determine the upper and lower boundaries of the cells, with a z-step interval of 0.1 μm for 3D Z-stack imaging. All datasets were saved in “nd2” format.

### 2.5 Fluorescence image-based reconstruction of cellular 3D STL structures

Raw “nd2” image datasets were batch-converted into sequential “tiff” files. Single-channel images corresponding to the cytoskeleton and nucleus were separated according to fluorescence channels and reconstructed into 3D volumetric datasets in MATLAB. The physical pixel size (μm/px) and z-axis step interval (μm) were defined to establish the spatial correspondence between voxel coordinates and physical spatial coordinates. Considering the fibrous high-noise characteristics of the cytoskeleton and the indistinct boundaries of the nucleus, multiple image enhancement and segmentation strategies were combined, including Frangi vesselness filtering, white top-hat transformation, contrast-limited adaptive histogram equalization (CLAHE), anisotropic diffusion filtering, adaptive thresholding, Otsu thresholding, and morphological operations. The segmented 3D binary volume data were subsequently smoothed using Gaussian filtering. Surface meshes were then generated using an isosurface extraction algorithm and exported in “stl” format. Following reconstruction, geometric parameters including vertex number, face number, surface area, and volume were calculated. Maximum intensity projection images along the z-axis were generated to verify segmentation quality. All intermediate processing results were preserved to ensure reproducibility of the reconstruction workflow.

### 2.6 Optimization of cellular 3D STL structures

The STL model was systematically optimized in SCDM. The STL model was imported, and geometric cleaning was performed: isolated fragments were deleted, and minor gaps and holes were repaired. Then, the surface was smoothed, and local high-curvature artifacts caused by fluorescence noise were removed while maintaining the overall volume and main geometric features. The Shrinkwrap function was used to wrap the complex surface body into a continuous closed surface to generate a continuous watertight solid body rather than a structure with internal voids, ensuring intact continuity of the cytoskeleton and favorable convergence during computational solving. To simulate the AFM indentation experiment, an assembly including the probe, cell, nucleus, and culture dish was constructed: a square substrate with a thickness of 0.1 μm was created; the cell was moved so that its lower surface was in contact without an initial gap with the upper surface of the substrate, simulating the adherent state; a spherical probe model with a radius of 0.25 μm was created and placed directly above the cell, and its lowest point was adjusted to be in perfect contact with the highest point of the cell. Finally, it was confirmed that there was no interference or initial penetration among the components, completing the assembly model suitable for FE analysis.

### 2.7 AFM nanoindentation experiments and force-curve analysis

QI mode was employed for nanoindentation measurements. The probe indented the cell surface at a speed of 50–88 μm/s, with a maximum indentation depth of 800 nm. For each cell, 128 × 128 spatial sampling points were collected, and at least 10 cells were measured in each experimental group. Raw force-curve data were batch-processed using JPK data analysis software. The processing workflow included probe calibration (spring constant and sensitivity), Gaussian smoothing (window size = 3), baseline correction, and contact-point identification. The indentation curves were fitted using the Hertz/Sneddon model with either a spherical or conical probe geometry. The Poisson’s ratio was set to 0.49, and the fitting range was defined as 20%–90% of the indentation segment to extract Young’s modulus values. Indentation depth under a specified reference force was also recorded for FE simulations. In addition, adhesion force was extracted from the retraction curve and defined as the maximum tensile force required for probe detachment from the cell surface.

### 2.8 FE modeling and numerical simulation

Based on the optimized 3D STL structure, a multibody contact FE model was established in ANSYS Workbench, including the probe, cytoskeleton, nucleus, and culture dish. Tetrahedral elements were used for meshing, with local mesh refinement applied to the probe–cell contact region. Mesh quality was evaluated according to element quality, aspect ratio, and Jacobian ratio. Three contact interfaces were defined: bonded contact between the nucleus and cytoskeleton to represent structural continuity, bonded contact between the basal cell surface and the substrate was used to approximate the constrained adherent state during AFM indentation, and frictionless contact between the probe and cytoskeleton with the “adjust to touch” option enabled. Material properties were assigned as follows. The probe was modeled as single-crystal silicon and treated as a rigid body (*E* = 162.7 × 10^6^ kPa, *ν* = 0.27). The culture dish was modeled as polystyrene with linear elastic behavior (*E* = 3.0 × 10^6^ kPa, *ν* = 0.34). The cell components were described using a Neo-Hookean hyperelastic constitutive model, in which the shear modulus and bulk modulus were converted from Young’s modulus values obtained from AFM experiments. For boundary conditions, the bottom surface of the substrate was fixed, and a normal displacement load was applied to the probe according to the indentation depth used in the AFM experiments. Large deformation and automatic time stepping were enabled during the solution process. After simulation, contact reaction force, total deformation, equivalent strain, and equivalent stress were extracted for analysis. In addition, a simplified model was constructed, in which the cell was represented as a hemispherical body with a radius of 10 μm and the probe as a rigid sphere with a radius of 0.25 μm. Simulations were performed using identical material models and loading conditions. The relative error of the contact force was calculated and compared with the analytical Hertz solution.

### 2.9 Statistical analysis

All experiments were independently repeated at least three times. Cell fluorescence images were quantitatively analyzed using ImageJ and NIS-Elements Viewer, and the data were summarized and statistically analyzed using GraphPad Prism. Quantitative data in this study, including cell mechanical parameters, geometric parameters, and numerical simulation results, were first subjected to the Shapiro–Wilk normality test and the Brown–Forsythe homogeneity of variance test. Normally distributed data were expressed as mean ± standard deviation. The independent-sample t-test was used for comparisons between two groups, and one-way ANOVA was used for comparisons among multiple groups (control group, low concentration group, and high concentration group). If the differences between groups were statistically significant, Tukey’s *post hoc* test was further conducted. For data that did not conform to a normal distribution or had unequal variances, non-parametric analysis was performed using the Kruskal–Wallis test. A significance level of *α* = 0.05 and *p* < 0.05 was considered statistically significant.

## 3 Results

### 3.1 Acquisition and characterization of CLSM fluorescence images

**FIGURE 2:**
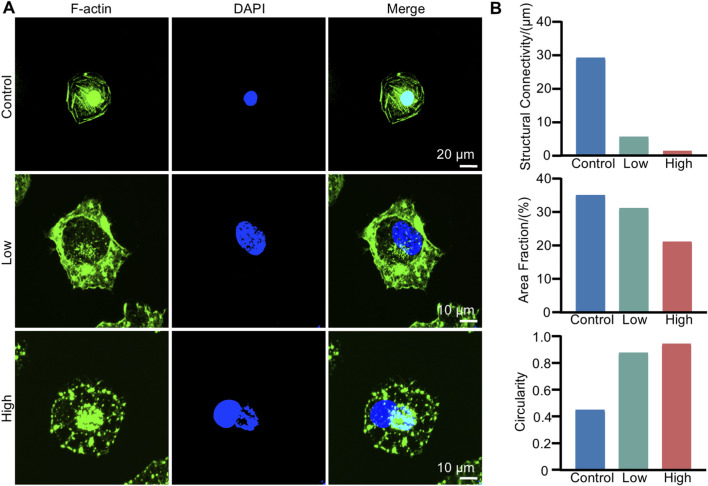
Changes and quantification of the cytoskeleton morphology of A549 cells after treatment with different concentrations of Cyto-D. **(A)** Representative fluorescence images of A549 cells; **(B)** Quantification of three indicators: structural connectivity, area fraction and circularity of the cytoskeleton. Note: **(A)** F-actin cytoskeleton was labeled with green fluorescence, and the nucleus was stained blue with DAPI. Calibrations (μm/pixel) for the control, low-concentration, and high-concentration groups: 0.35, 0.09, and 0.17 μm/pixel; **(B)** Structural connectivity reflects the continuity of the cytoskeletal network; area fraction represents the proportion of image area occupied by cytoskeletal structures; circularity describes the degree to which structures approach a circular morphology, with higher values indicating more condensed and aggregated cytoskeletal organization.

Fluorescence imaging of A549 cells treated with different concentrations of Cyto-D revealed concentration-dependent cytoskeletal remodeling (Supplementary Figure 1). Control cells spread well and had clear boundaries, were elongated in shape, with a continuous and uniformly distributed radial cytoskeletal network; filaments were straight with clear polarity, and the nuclei were regularly round or oval, positioned centrally within the cytoskeleton. After low-concentration Cyto-D treatment, the number of stress fibers decreased and contracted, the cytoskeleton aggregated into rod-like structures, and some cell edges became wrinkled. In the high-concentration group, the cytoskeleton was severely depolymerized, the radial filaments almost disappeared, dense spots or spherical structures formed around the nucleus, cells were significantly contracted with reduced spreading area, and nuclei showed eccentric distribution (Figure 2A). Quantitative analysis further confirmed: Structural connectivity dropped from 29.09 μm in the control group to 5.53 μm and 1.34 μm in the low and high concentration groups (a 21-fold decrease). The area fraction decreased from 34.89% to 20.97%. Meanwhile, circularity rose from 0.45 to 0.94. These findings show that higher Cyto-D concentrations force the A549 cytoskeleton to shift from an intact fibrous network to fragmented, aggregated states. It is clear that the cytoskeleton is the primary driver for maintaining cellular spatial morphology and internal structural stability (Figure 2B).

### 3.2 3D STL structural reconstruction of cells based on MATLAB image processing

**FIGURE 3:**
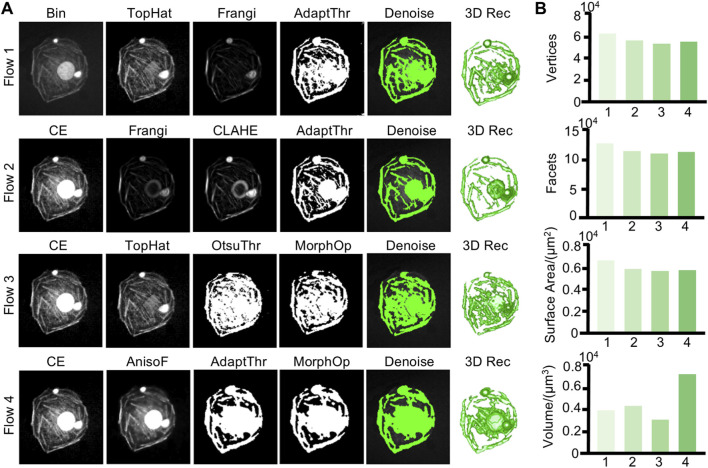
Reconstruction and geometric parameter quantification of cytoskeletal structures in control group A549 cells based on MATLAB image processing. **(A)** Maximum intensity projection images of the preprocessed four parallel processes of the cytoskeleton image in the cropping mode with a sensitivity of 0.6; **(B)** Geometric parameter quantification of the four parallel processes. Note: Abbreviations: AdaptThr, Adaptive thresholding; AnisoF, Anisotropic diffusion filtering; Bin, Binarization; CE, Contrast Enhancement; CLAHE, Contrast-Limited Adaptive Histogram Equalization; Den, Denoising; Frangi, Frangi vesselness filtering; MorphOp, Morphological operations; OtsuThr, Otsu thresholding; TopHat, White top-hat transformation; 3D Rec, 3D Reconstruction.

**FIGURE 4:**
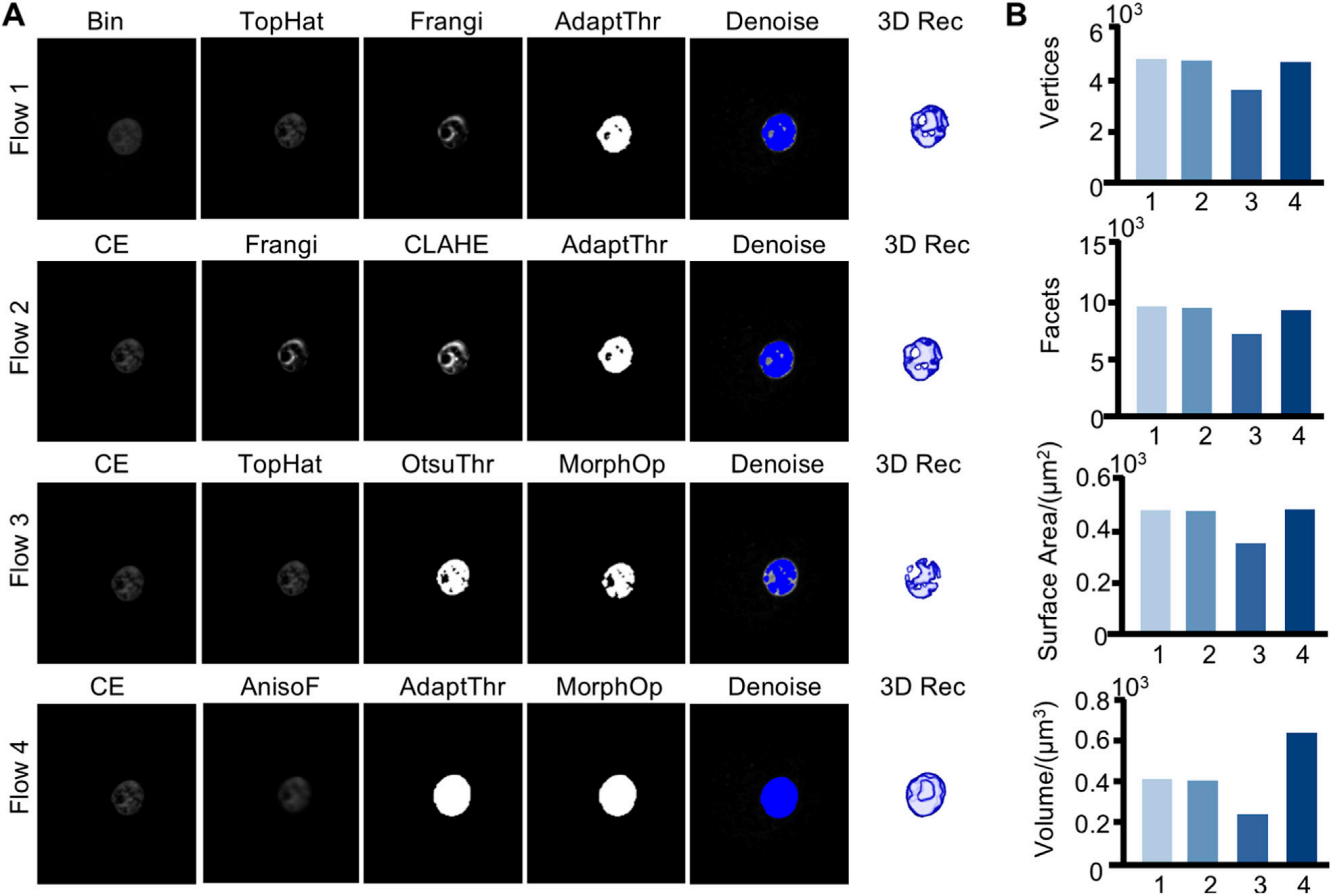
Reconstruction and geometric parameter quantification of nuclear structures in control group A549 cells based on MATLAB image processing. **(A)** Maximum intensity projection images of the preprocessed four parallel processes of the nuclear image in the cropping mode with a sensitivity of 0.6; **(B)** Geometric parameter quantification of the four parallel processes. Note: Abbreviations: AdaptThr, Adaptive thresholding; AnisoF, Anisotropic diffusion filtering; Bin, Binarization; CE, Contrast Enhancement; CLAHE, Contrast-Limited Adaptive Histogram Equalization; Den, Denoising; Frangi, Frangi vesselness filtering; MorphOp, Morphological operations; OtsuThr, Otsu thresholding; TopHat, White top-hat transformation; 3D Rec, 3D Reconstruction.

Through a self-developed MATLAB program, the optimal reconstruction path was selected from four parallel processing procedures, successfully reconstructing the 3D STL surface models of the A549 cytoskeleton and nucleus while preserving subtle surface morphological features and asymmetric nuclear distributions. To balance noise suppression and shape fidelity, the cropping mode at a sensitivity of 0.6 was chosen as the baseline (Supplementary Figure 2). The cytoskeleton structure with optimal connectivity was extracted through Process 1 (contrast enhancement → white top-hat transformation → Frangi filtering → adaptive thresholding → denoising) (Figure 3A). At a sensitivity of 0.6, Process 1 generated 63,076 vertices, 129,282 mesh faces, a surface area of 6,682.22 μm^2^, and a volume of 3,944.57 μm^3^, yielding the highest numbers of vertices and facets, as well as the largest surface area (Figure 3B). For the nucleus, Process 4 (contrast enhancement → anisotropic diffusion → adaptive thresholding → morphological opening → denoising) generated the smoothest STL surface models while preserving the overall nuclear curvature characteristics (Figure 4A), with 4,652 vertices, 9,312 facets, a surface area of 484.01 μm^2^, and a volume of 639.71 μm^3^ (Figure 4B). Therefore, subsequent cytoskeleton modeling adopted Process 1, and nucleus modeling adopted Process 4.

**FIGURE 5:**
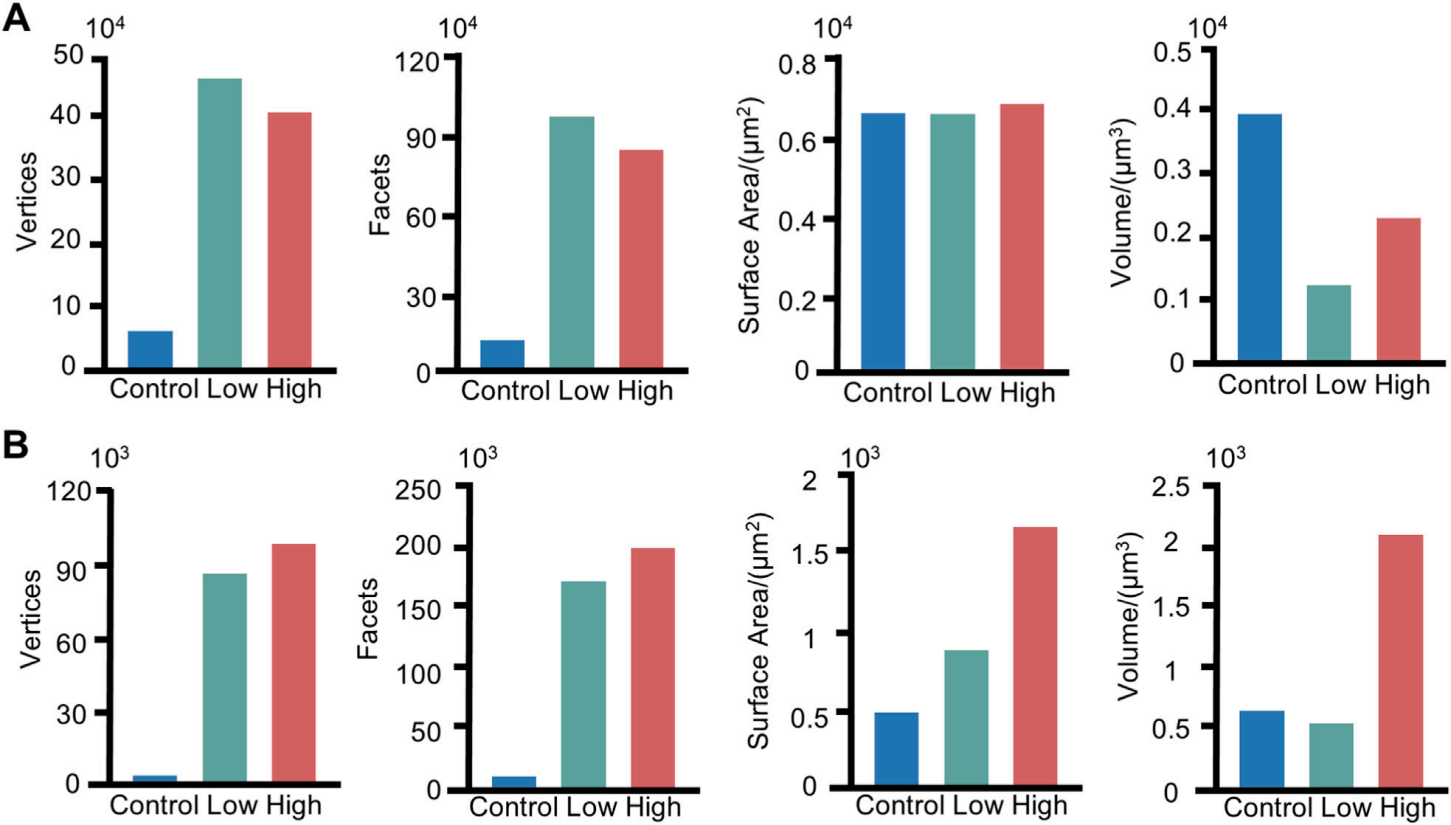
Reconstruction and geometric parameter quantification of cytoskeleton and nuclear structures in three groups of A549 cells based on MATLAB image processing. **(A)** Comparison of 3D reconstruction geometric parameters of the cytoskeleton from processes one at sensitivity 0.6 under cropping mode in each group; **(B)** Comparison of 3D reconstruction geometric parameters of the nucleus from processes 4 at sensitivity 0.6 under cropping mode in each group.

The 3D morphological parameters of the cytoskeleton differed significantly under different treatments. In the low concentration group, the number of vertices/facets surged to 461,144/981,888 (approximately a 7.31-fold increase), while the volume decreased to 1,235.56 μm^3^ (approximately 31.32% decrease). In the high concentration group, the number of vertices/facets was 408,481/851,556, and the volume was 2,301.31 μm^3^. Surface area remained relatively stable among the three groups (approximately 6,654–6,918 μm^2^) (Figure 5A). Regarding the nucleus, the control group had 4,652/9,312 vertices/facets with a surface area of 484.01 μm^2^. In the high concentration group, this increased to 97,983/201,404 (an approximately 21.06-fold increase), with a surface area of 1,668.94 μm^2^ (increased by approximately 3.45 times). The nuclear volume of the control group was 639.71 μm^3^, which decreased to 527.42 μm^3^ in the low concentration group, and increased to 2,103.37 μm^3^ (an approximately 3.29-fold increase) in the high concentration group (Figure 5B). These results reveal the asynchronous nature of Cyto-D-induced cytoskeleton disassembly and nuclear morphological remodeling.

### 3.3 SCDM-based optimization of cellular 3D STL surface models

**FIGURE 6:**
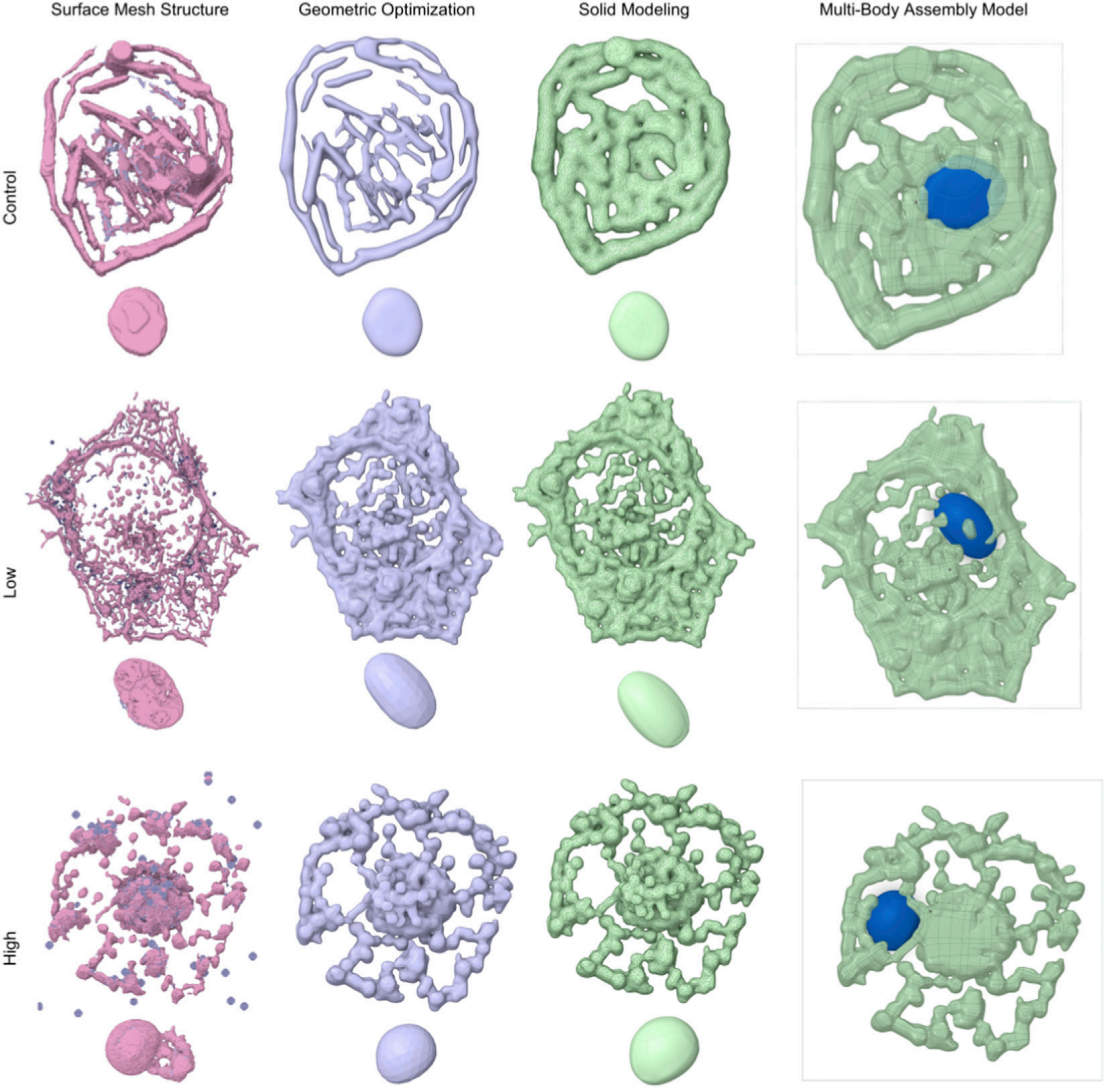
Defect repair, solid optimization and assembly of 3D sheet structures for three groups of cells based on SCDM.

The reconstructed 3D surface meshes (with over 10,000 facets for each model) contained inherent defects, including isolated fragments, disconnected facets and non-closed surfaces, which made them unsuitable for direct FE analysis. After structural optimization via SCDM, non-manifold edges, jagged artifacts and internal holes were eliminated, and discrete surface models were converted into continuous closed solid models. Both the cytoskeleton and the nucleus were transformed from surface bodies to solid bodies in the structural tree, satisfying the requirements for contact definition and FE computation. Geometric inspection and interference detection verified the absence of penetration or gaps within the assembly. A four-component multi-body contact model consisting of the probe, cytoskeleton, nucleus and culture dish was finally established, realizing precise nuclear embedding and rational contact relationships among all components (Figure 6).

### 3.4 Quantitative characterization of AFM nanomechanical properties

**FIGURE 7:**
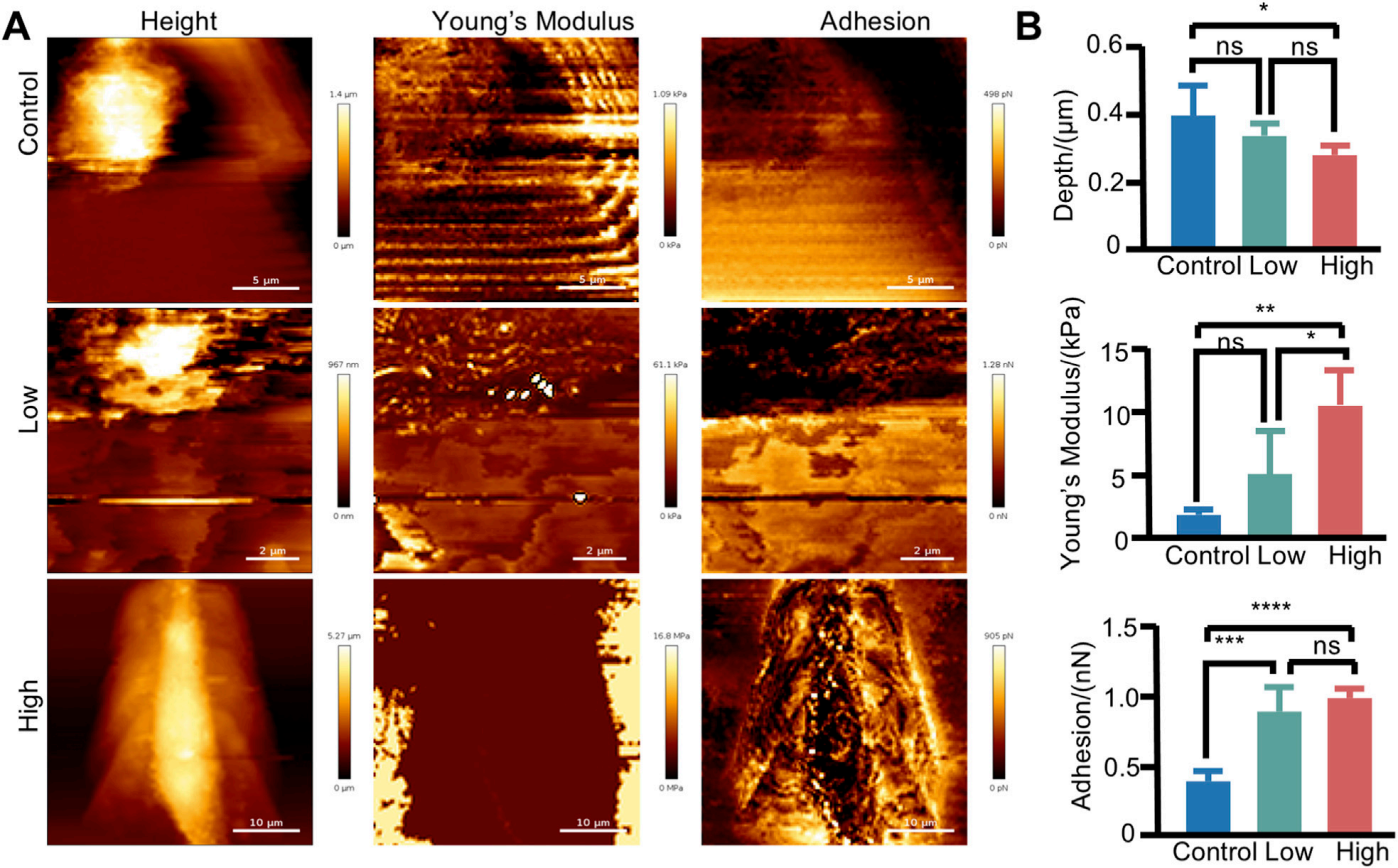
Quantitative nanomechanical characterization of A549 cells after treatment with different concentrations of Cyto-D. **(A)** Cell morphology and force spectroscopy maps; **(B)** Comparative analysis of depth, Young’s modulus, and adhesion.

Based on the high-throughput force–indentation curves obtained from AFM nanoindentation experiments (a total of 30 cell samples and 5.4 × 10^5^ force curves), the Young’s modulus and adhesion force were extracted after processing with the JPK Data Processing software. The median values of each parameter were then calculated using MATLAB to serve as representative values for the cells (Figure 7A). After data cleaning, the number of effective force curves in the control group was 60,586 (with an effective detection point percentage of 92.4% ± 4.4%), in the low concentration group was 50,881 (89.0%), and in the high concentration group was 50,720 (77.4% ± 19.1%). There were significant differences in the mechanical parameters of cells treated with different concentrations of Cyto-D (*p* < 0.05). The Young’s modulus increased in a concentration-dependent manner: that of the control group was 1.81 ± 0.40 kPa, that of the low concentration group increased to 5.15 ± 3.45 kPa, and that of the high concentration group further increased to 10.54 ± 2.74 kPa. The adhesion force also showed a similar trend: the control group was 0.39 ± 0.06 nN, the low concentration group was 0.89 ± 0.17 nN, and the high concentration group was 0.99 ± 0.06 nN (*p* < 0.05). The indentation depth (δ) gradually decreased from approximately 0.36 μm in the control group to 0.34 μm in the low concentration group and 0.29 μm in the high concentration group, indicating that the deformation ability of cells under the same load decreased with the increase in Cyto-D concentration (Figure 7B).

### 3.5 FE simulation and numerical verification

**FIGURE 8:**
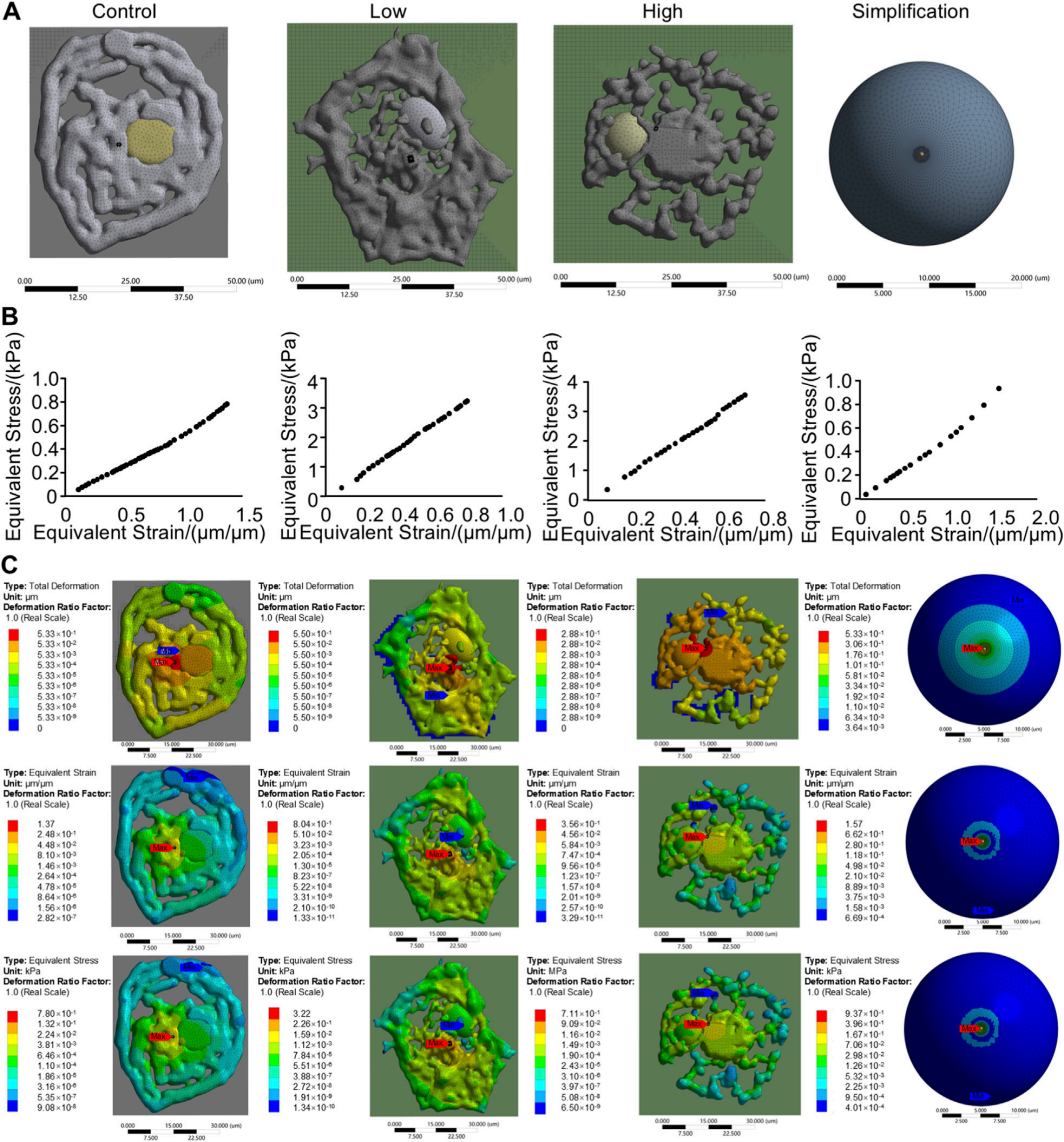
Mesh generation and simulation result comparison of four groups of FE models. **(A)** Mesh generation; **(B)** Stress-strain curves; **(C)** Contour plots of total deformation, equivalent strain and equivalent stress.

The indentation depth was *δ* = 0.3–0.4 μm, and the simplified cell radius was *Rc* = 10 μm (*δ* ≪ *Rc*, *ε* = 0.03–0.04 < 5%), which is consistent with the small deformation assumption of Hertz contact theory. Mesh quality evaluation indicated good element quality and numerical stability (Figure 8A; Supplementary Figures 3, 4, Supplementary Table 1). In the contact setup, the initial states of the nucleus-cytoskeleton, cytoskeleton-culture dish, and probe-cytoskeleton interfaces had no penetration or gaps, satisfying physical validity (Supplementary Figure 5, Supplementary Table 2). All four FE models, including three image-based cell models and one simplified model, exhibited approximately linear stress–strain relationships during probe displacement loading, indicating that the cellular mechanical response under shallow indentation and quasi-static conditions could be reasonably approximated as linear elastic behavior (Figure 8B). The image-based models demonstrated pronounced structure-dependent mechanical responses characterized by nonuniform and asymmetric stress transmission. Compared with the cytoskeletal region, the nucleus exhibited lower deformation, strain, and stress levels, while still participating in progressive force transmission and forming a continuous mechanical gradient within the cell. No abrupt mechanical discontinuity was observed at the nucleus–cytoskeleton interface; instead, the stress distribution transitioned smoothly across the interface, suggesting a buffering effect that may contribute to intracellular structural stability. Mechanical responses also showed clear Cyto-D concentration dependence. In the control group, deformation was mainly localized beneath the probe, indicating effective load redistribution by the intact cytoskeletal network. With increasing Cyto-D concentration, the deformation and stress distributions gradually shifted from localized concentration to more diffuse patterns, accompanied by fragmentation of stress transmission pathways and increased stress accumulation around the nucleus. These findings suggest that cytoskeletal depolymerization plays a major role in regulating intracellular intracellular force propagation. In contrast, the simplified model consistently exhibited idealized axisymmetric stress distributions and failed to capture the mechanical heterogeneity associated with cytoskeletal remodeling (Figure 8C).

## 4 Discussion

The cytoskeleton not only maintains cellular morphology but also serves as a key mechanical framework for sensing external mechanical stimuli and transmitting them throughout the cell (Discher et al., 2005; Haspinger et al., 2021). Previous studies have shown that cytoskeletal remodeling can substantially alter cellular mechanical properties and influence intracellular stress transmission as well as nuclear mechanics (Martino et al., 2018; Yang et al., 2023). Therefore, mechanically representative models based on realistic cellular structures are important for understanding cellular mechanical behavior and its biological functions. In this study, CLSM fluorescence imaging, AFM nanoindentation, and FE simulations were integrated to investigate Cyto-D-induced cytoskeletal remodeling and the associated mechanical responses of A549 cells.

CLSM observations revealed that F-actin stress fibers in control cells formed a continuous radial network with an organized tension-bearing architecture. With increasing Cyto-D concentration, F-actin polymerization was progressively inhibited, resulting in stress-fiber disruption and centripetal contraction of the cytoskeleton. Consequently, the cytoskeletal organization transitioned from a continuous filamentous network to discontinuous rod-like or punctate structures (Elblová et al., 2024; Fujimoto and Nakazawa, 2024; Zhang H. et al., 2023). These structural alterations were accompanied by cellular contraction, reduced spreading area, and eccentric nuclear positioning, indicating that the inhibition of F-actin polymerization compromises cytoskeletal support and promotes cellular structural remodeling (Fink et al., 2024; Holle et al., 2018; Jia and Liu, 2014; Zhang M. et al., 2023).

AFM nanoindentation measurements further demonstrated significant changes in cellular mechanical properties following Cyto-D treatment. Increasing Cyto-D concentration was associated with reduced indentation depth and increased apparent Young’s modulus and adhesion force. These changes may be related to enhanced membrane–cytoskeleton coupling and local stress-fiber reorganization (Long et al., 2024; Yu and Wei, 2023; Lv et al., 2021; Song et al., 2019; Fan et al., 2022; Kahle et al., 2022; Lü et al., 2023; Heath et al., 2021; Witherel et al., 2021). Although Cyto-D disrupts the global tensional network and induces severe cellular contraction, our extraction algorithm for batch processing AFM force curves in MATLAB was configured to exclusively encompass valid data points marked with ‘Include’ flags, specifically targeting the P70 to P90 percentile threshold range of the aggregate data. This extraction criterion, independent of the global sample distribution, precisely captures the mechanical signatures of the localized, high-stiffness actin aggregates formed subsequent to the collapse of the cytoskeletal network. Furthermore, existing studies have reported pronounced apparent stiffening phenomena under specific experimental conditions following actin depolymerization, which can be attributed to cytoskeletal collapse, cellular contraction, increased membrane density, and altered membrane-cytoskeleton interactions (Gruber et al., 2023). It is also well established that cellular mechanical readouts are highly sensitive to the specific AFM instrumentation and parameters employed (Yang et al., 2021). Consequently, the extracted high-percentile (P70–P90) modulus reflects the localized mechanical response of the structurally reorganized cell under the current AFM testing conditions, rather than a uniform global cellular softening.

FE simulations showed that the image-based cell models more accurately represented intracellular mechanical transmission than the simplified benchmark model. In the image-based models, equivalent stress was primarily concentrated beneath the probe–cell contact region and propagated inward along cytoskeletal structures, indicating that the cytoskeleton serves as the principal pathway for load transfer. Although the nucleus exhibited lower deformation levels than the surrounding cytoskeleton, it remained involved in continuous force transmission. Biologically, the cytoskeleton functions as a primary structural buffer; its higher stress load reflects its capacity to dissipate external mechanical energy, thereby shielding the nucleus from localized stress concentrations. As Cyto-D concentration increased, stress transmission gradually shifted from localized concentration to a more diffuse distribution, accompanied by fragmentation of transmission pathways and enhanced stress accumulation around the nucleus. These findings indicate that image-based models can capture the influence of structural heterogeneity and spatial complexity on cellular mechanical behavior, whereas simplified models cannot adequately represent these features.

However, several limitations of the present study should be acknowledged. Cells are inherently viscoelastic materials with rate-dependent mechanical responses (Efremov et al., 2020; Mendiola et al., 2026), while only quasi-static indentation and hyperelastic constitutive law were adopted here to ignore time-dependent viscoelastic behaviors. Our model oversimplifies intricate cytoplasmic structures and organelles into homogeneous continua, assigning identical uniform material properties to both nuclear and extranuclear regions and neglecting the spatial heterogeneity of cytoskeletal density and mechanics. In addition, actomyosin contraction-induced cellular prestress was not integrated into the simulation framework; proper introduction of initial stress field could rearrange intracellular stress distribution and narrow numerical-experimental deviation, which matters considerably because aberrant mechanical coupling between subcellular compartments drives maladaptive cellular phenotypes during aging, oncogenesis and vascular remodeling (Brookes et al., 2023). Although the nucleus and cytoskeleton were reconstructed as separate geometric domains, isotropic Hookean material model was applied without capturing cytoskeletal fiber orientation, anisotropy and regional density variation. Besides, all simulations merely covered quasi-static shallow indentation, whereas cellular mechanical signatures and internal stress distribution would greatly differ under physiological stimuli including fluid shear and substrate stretching. To leverage the segmented 3D structural information from fluorescence images and achieve high-fidelity cell modeling, future work will combine viscoelastic formulations, multiscale frameworks and fiber-reinforced anisotropic constitutive models (e.g., modified Holzapfel-Gasser-Ogden model) to reproduce compartment-specific heterogeneous mechanical responses.

## 5 Conclusion

From a methodological perspective, this study achieved morphologically accurate reconstruction of three-dimensional STL models from CLSM fluorescence image stacks and established a multibody contact FE framework through SCDM-based topological repair and geometric optimization. The model was validated using mechanical parameters obtained from AFM QI measurements, thereby reducing the limitations associated with the geometric simplifications of conventional spherical or ellipsoidal cell models. The resulting framework demonstrated improved geometric fidelity and robust numerical convergence, providing a feasible approach for integrating fluorescence imaging with FE analysis in cell mechanics research and a methodological reference for investigating the relationship between cytoskeletal organization and cellular mechanical behavior. More broadly, the integration of imaging-derived geometries with computational biomechanics provides an effective strategy for bridging cellular structure and mechanical function, which may facilitate future studies in mechanobiology, disease modeling, and cell engineering.

## Data Availability

The raw data supporting the conclusions of this article will be made available by the authors, without undue reservation.
