# Supplementary material for: Construction and simulation-experimental characterization of a fluorescence image-based finite element model for cell mechanics
Source: Front Bioeng Biotechnol. 2026 Jul 22;14:1894529. doi: 10.3389/fbioe.2026.1894529 (PMC13438368; doi:10.3389/fbioe.2026.1894529)
Supplement: Supplementary file 1 [file Supplementaryfile1.docx]

Supplementary Material

**Supplementary Figure 1.** Changes in A549 cells structure under different Cyto-D concentration interventions.

**Note:** Calibrations (μm/pixel) for the control, low-concentration, and high-concentration groups: 0.17, 0.43, and 0.29.

**Supplementary Figure 2.** MATLAB image preprocessing, segmentation and 3D cytoskeletal structure reconstruction.

**Supplementary Figure 3.** Statistical information on mesh generation of the FE model.

**Supplementary Figure 4.** Quality on mesh generation of the FE model.

**Supplementary Figure 5.** Contact settings for the FE Model of control group cells.

**Supplementary Tab 1.** Detailed table of FE Model mesh quality

| Group | Structure | Nodes | Elements | Element Quality | Aspect Ratio | Jacobian Ratio |
| --- | --- | --- | --- | --- | --- | --- |
| Control | Cytoskeleton | 200002 | 135153 | 0.7979 | 1.9989 | 0.9771 |
| Control | Nucleus | 6415 | 4038 | 0.8253 | 1.9283 | 0.9691 |
| Control | Culture Dish | 7961 | 2586 | 0.9999 | 1.0091 | 0.9974 |
| Control | Probe | 171 | 85 | 0.9263 | 1.3330 | 1.0000 |
| Control | FE Model | 214549 | 141862 | 0.8025 | 1.9784 | 0.9772 |
| Low | Cytoskeleton | 225493 | 151129 | 0.7973 | 2.0187 | 0.9744 |
| Low | Nucleus | 4084 | 2517 | 0.8351 | 1.9112 | 0.9700 |
| Low | Culture Dish | 10300 | 3357 | 0.9995 | 1.0152 | 0.9958 |
| Low | Probe | 195 | 97 | 0.9166 | 1.3675 | 1.0000 |
| Low | FE Model | 240072 | 157100 | 0.8023 | 1.9951 | 0.9748 |
| High | Cytoskeleton | 153758 | 101347 | 0.7936 | 2.0377 | 0.9663 |
| High | Nucleus | 4284 | 2679 | 0.8295 | 1.9251 | 0.9693 |
| High | Culture Dish | 8966 | 2917 | 1.0000 | 1.0000 | 1.0000 |
| High | Probe | 195 | 97 | 0.9166 | 1.3675 | 1.0000 |
| High | FE Model | 167203 | 107040 | 0.8002 | 2.0060 | 0.9674 |
| Simplification | Cell | 80589 | 55394 | 0.8282 | 1.8715 | 0.9959 |
| Simplification | Probe | 163 | 81 | 0.9034 | 1.4159 | 1.0000 |
| Simplification | FE Model | 80752 | 55475 | 0.8283 | 1.8708 | 0.9959 |

**Supplementary Tab 2.** Contact status detection details table

| Group | Contact Region | Contact Type | Status | Contact Count | Penetration (μm) | Gap (μm) |
| --- | --- | --- | --- | --- | --- | --- |
| Control | Nucleus to Cytoskeleton | Bonded | Close | 76 | 8.87×10^-14^ | 0 |
| Control | Cytoskeleton to Culture Dish | Bonded | Close | 3074 | 1.93×10^-14^ | 0 |
| Control | Cytoskeleton to Probe | Frictionless | Close | 1 | 3.19×10^-15^ | 0 |
| Low | Nucleus to Cytoskeleton | Bonded | Close | 28 | 0 | 0 |
| Low | Cytoskeleton to Culture Dish | Bonded | Close | 2774 | 0 | 0 |
| Low | Cytoskeleton to Probe | Frictionless | Close | 1 | 1.10×10^-15^ | 0 |
| High | Nucleus to Cytoskeleton | Bonded | Close | 136 | 0 | 0 |
| High | Cytoskeleton to Culture Dish | Bonded | Close | 1696 | 0 | 0 |
| High | Cytoskeleton to Probe | Frictionless | Close | 1 | 5.60×10^-17^ | 0 |
| Simplification | Cell to Probe | Frictionless | Close | 2 | 1.66×10^-20^ | 0 |

**Supplementary Figure 6.** Representative force-indentation curves of cells obtained by AFM quantitative imaging mode.

**Note:** (A) Curve from non-cellular blank background at the scanning zero-coordinate point (bottom-left of frame). (B) Curve from the brightest single-cell site (No. 5571) in the scanning area. Representative vertical deflection-indentation height curves recorded during AFM indentation tests. The light cyan curve corresponds to the probe extending (Extend) toward the cell surface, while the dark blue curve represents the retraction (Retract) process after indentation. The vertical axis denotes vertical deflection converted into contact force (nN), and the horizontal axis presents the uncalibrated Z-scan height (μm). AFM testing parameters: indentation depth 0.5 μm, indentation speed 88 μm/s, target setpoint force 2.213 nN, data acquisition rate 88 kHz. The hysteresis gap between extend and retract curves reflects the viscoelastic deformation behavior of cytoskeleton and cell nucleus under mechanical compression.
